# Influences of Head Motion Regression on High-Frequency Oscillation Amplitudes of Resting-State fMRI Signals

**DOI:** 10.3389/fnhum.2016.00243

**Published:** 2016-05-26

**Authors:** Bin-Ke Yuan, Yu-Feng Zang, Dong-Qiang Liu

**Affiliations:** ^1^Center for Cognition and Brain Disorders, Hangzhou Normal UniversityHangzhou, China; ^2^Zhejiang Key Laboratory for Research in Assessment of Cognitive Impairments, Hangzhou Normal UniversityHangzhou, China; ^3^Research Center of Brain and Cognitive Neuroscience, Liaoning Normal UniversityDalian, China

**Keywords:** resting-state fMRI, high-frequency oscillations, fluctuation amplitude, head motion, eyes open, eyes closed

## Abstract

High-frequency oscillations (HFOs, >0.1 Hz) of resting-state fMRI (rs-fMRI) signals have received much attention in recent years. Denoising is critical for HFO studies. Previous work indicated that head motion (HM) has remarkable influences on a variety of rs-fMRI metrics, but its influences on rs-fMRI HFOs are still unknown. In this study, we investigated the impacts of HM regression (HMR) on HFO results using a fast sampling rs-fMRI dataset. We demonstrated that apparent high-frequency (∼0.2–0.4 Hz) components existed in the HM trajectories in almost all subjects. In addition, we found that individual-level HMR could robustly reveal more between-condition (eye-open vs. eye-closed) amplitude differences in high-frequency bands. Although regression of mean framewise displacement (FD) at the group level had little impact on the results, mean FD could significantly account for inter-subject variance of HFOs even after individual-level HMR. Our findings suggest that HM artifacts should not be ignored in HFO studies, and HMR is necessary for detecting HFO between-condition differences.

## Introduction

Resting-state functional magnetic resonance imaging (rs-fMRI) has been widely used to investigate spontaneous brain activity. Most rs-fMRI studies have focused on the low-frequency (usually below 0.1 Hz) band of signal (Biswal et al., 1995; Lowe et al., 1998; Cordes et al., 2000, 2001; Greicius et al., 2003). High-frequency oscillations (HFOs, >0.1 Hz) are usually discarded probably because HFOs are vulnerable to physiological noise, and conventional techniques restrict exploration of higher frequency bands. However, HFOs are drawing more and more attention. Commonly known resting-state networks (e.g., default mode network, visual network, and motor network) can be observed at frequency bands higher than conventional ones (Boubela et al., 2013; Boyacioglu et al., 2013; Lee et al., 2013; Gohel and Biswal, 2014; Kalcher et al., 2014). The fluctuation amplitude of HFOs exhibits organized spatial patterns (Baria et al., 2011; Kalcher et al., 2014). Moreover, high-frequency changes have been detected between different brain states (Yuan et al., 2014) and in some diseases (Malinen et al., 2010; Baliki et al., 2011; Hong et al., 2013; Otti et al., 2013; Wang et al., 2015; Yu et al., 2015; Yue et al., 2015). For example, Malinen et al. (2010) found that patients with chronic spinal and limb pain showed greater high-frequency (0.12–0.25 Hz) fluctuation amplitude in the bilateral insular cortices and anterior cingulate cortex compared with healthy controls. Patients with chronic back pain have demonstrated significantly increased HFOs (0.12–0.20 Hz) in the insular cortex, medial prefrontal cortex, and posterior cingulate cortex (Baliki et al., 2011). These findings imply that HFOs of rs-fMRI signals may provide additional information about organization of the resting human brain beyond low-frequency oscillations (LFOs).

In recent years, fast imaging techniques have enabled us to acquire whole-brain fMRI data in less than 1 s (Hennig et al., 2007; Feinberg et al., 2010; Boyacioglu and Barth, 2013). An increasing number of studies have begun to investigate rs-fMRI HFOs using fast temporal sampling rates (Boubela et al., 2013; Boyacioglu et al., 2013; Lee et al., 2013; Yuan et al., 2014). Our previous study has confirmed the superiority of a fast sampling rate in that it can improve sensitivity in detecting between-condition differences in HFOs and suppress artifacts (Yuan et al., 2014). However, other methodological issues remain unclear. HFOs are more susceptible than LFOs to physiological activities. Thus, denoising is a critical problem for HFO studies. Regression of covariates including white matter (WM), cerebrospinal fluid (CSF), as well as head motion (HM) is typically a routine step for the majority of LFO studies. As for HFO studies, the necessity of WM and CSF regression is evident, but the influences of HM on HFO results remain unclear. Previous HFO studies differ greatly in how they treated the HM effects. Some regressed out the HM covariates, whereas others did not (Malinen et al., 2010; Boubela et al., 2013; Boyacioglu et al., 2013; Hong et al., 2013; Lee et al., 2013; Otti et al., 2013), which makes it difficult to compare results among different studies. Hence, there is a need to evaluate the influence of HM regression (HMR) on HFO results.

In this study, we used the same short-TR dataset collected in our previous study (Yuan et al., 2014) to investigate the following questions. First, we wondered whether the HM trajectories contain conspicuous high-frequency components. Second, we were interested in how HMR affects detection of HFO amplitude changes, since HFO amplitude has been used recently to detect abnormalities in some brain disorders. In particular, we examined HFO amplitude differences between eyes-open (EO) and eyes-closed (EC) resting states, and we compared results with and without HMR. To characterize comprehensively the influences of HMR on HFO results, we assessed the performances of both individual- and group-level HMR approaches (Yan et al., 2013a).

## Materials and Methods

### Participants and Data Acquisition

The dataset used in this study was from INDI Retrospective Data Sharing Samples ^1^ (HNU short TR: Short-TR EO/EC Resting State fMRI Data), which was collected in our previous study (Yuan et al., 2014). Forty-six healthy adults (24.8 ± 1.7 years, range 22–32; 23 females) were enrolled. Each participant gave written informed consent. They were screened with a questionnaire to ensure no history of brain injury, neurological illness, or psychiatric disorders. The study was approved by the ethics committee of the Center for Cognition and Brain Disorders, Hangzhou Normal University.

**FIGURE 1:**
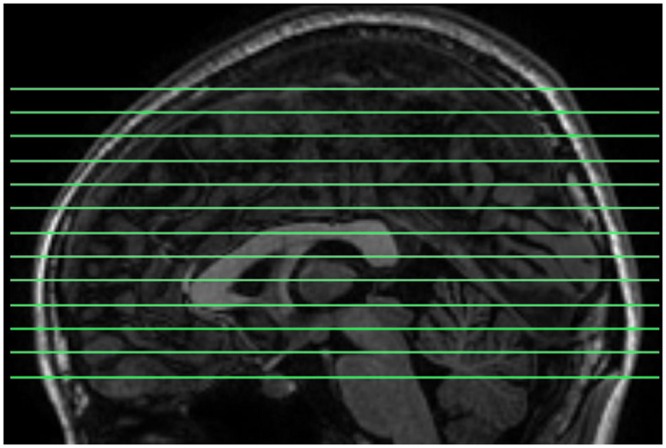
**The slice positions for short-TR rs-fMRI data acquisition.** TR = 400 ms, total number of slices = 13, and thickness/gap = 6 mm/1 mm. Most parts of the cerebrum were covered.

Magnetic resonance images were acquired using a GE Discovery MR-750 3.0 T scanner (GE Medical Systems, Waukesha, WI, USA) in the Center for Cognition and Brain Disorders of Hangzhou Normal University. The participants lay supine with the head snugly fixed by straps and foam pads to minimize movement. The procedures for collecting data were as follows. First, an EC resting state session was scanned using a conventional sampling rate (TR = 2 s). This session was acquired for another purpose and not analyzed here. Then, a 3D T1-weighted image was scanned using a spoiled gradient-recalled (SPGR) pulse sequence (176 sagittal slices, thickness = 1 mm, TR = 8100 ms, TE = 3.1 ms, flip angle = 8°, FOV = 250 mm × 250 mm). Two short-TR rs-fMRI sessions, EO and EC, were scanned. The order of the EO/EC conditions was counterbalanced across subjects. Each of the two sessions lasted 8 min, consisting of 1200 volumes. The scanning parameters were: TR = 400 ms, TE = 15 ms, flip angle = 30°, thickness/gap = 6/1 mm, FOV = 240 mm × 240 mm, matrix = 64 × 64, 13 axial slices. In this study, we aimed to investigate HFOs using a fast sampling rate and within the whole brain. However, for conventional techniques, increasing TR decreases the brain coverage. To meet both criteria of fast sampling rate and whole brain coverage, we tuned other parameters. First, we increased the slice thickness to 7 mm. Second, we shortened the TE to increase the maximal number of slices to 13, because there is a trade-off between TE and the maximum number of slices for our scanner. The resulting slice position covered most parts of the cerebrum (**Figure 1**). In addition, 13 slices of T1 images with the same slice positions as the short-TR fMRI data were obtained, using T1-weighted fluid-attenuated inversion recovery (FLAIR) pulse sequence (thickness/gap = 6/1 mm, TR = 2382 ms, TE = 25 ms, flip angle = 90°, FOV = 240 mm × 240 mm, matrix = 512 × 512).

During all of the resting state sessions, subjects were instructed to keep as motionless as possible, to try to stay relaxed, not to think of anything particular, and not to fall asleep while the scanner room was kept dim during scanning. At the end of each scanning session, one experimenter talked to the subjects, and all subjects confirmed that they did not fall asleep during the preceding session.

### Data Preprocessing

The data underwent the same preprocessing steps as described in our previous work (Yuan et al., 2014). Briefly, for the functional images, the first 50 volumes (20 s) were discarded. Slice timing and HM correction were performed. Six HM time series were obtained. No subject had HM with more than 2.0 mm maximum displacement in any direction of *x, y*, and *z* or 2° of any angular motion throughout the scan. The 3D T1 image was first co-registered with the multi-slice T1 images, and then normalized to the Montreal Neurological Institute (MNI) space. Consequently, a transforming matrix from the original space to the standard space was obtained. The transformation matrix was then directly applied to the functional images. Finally, we removed the temporal linear trend from the spatially normalized functional data. These preprocessing procedures were performed using SPM8^2^ (Statistical Parameter Mapping) and REST^3^ (Song et al., 2011) packages. We calculated the power spectrum of HM time series for each subject and each condition.

### Nuisance Covariates Regression

High-frequency oscillations are often contaminated by physiological noise (Cordes et al., 2001). In addition, our previous study demonstrated that nuisance covariates regression could reduce artificial between-condition differences in WM in high-frequency bands (Yuan et al., 2014). Hence, in this study, several nuisance covariates were regressed out from the data. These covariates included: (1) the averaged time series within the WM ROIs provided by the REST software, (2) the averaged time series of the CSF signal within the ventricle ROIs provided by the REST software, (3) the averaged signal within a spherical ROI in the suprasellar cistern (SC) (MNI coordinate: *x* = -6, *y* = -2, *z* = -17; radius = 6 mm) adjacent to the Circle of Willis.

Previous work (Lund et al., 2006) has shown that influences of cardiac and respiratory activities have different spatial distributions. Cardiac-induced noise is dominant near larger vessels (e.g., the medial cerebral artery and Circle of Willis), while respiratory-induced noise occurs near the large veins and in the ventricles. The CSF ROI used in the present study mainly covers ventricles. To capture fully and remove the temporal dynamics of physiological noises, we added the SC covariate, in addition to the WM and CSF covariates, to the regression model. Our previous work has shown that HFO amplitude results with global signal regression (GSR) exhibited a noisy pattern compared with those without GSR (Yuan et al., 2014). Thus, we did not perform GSR in the current study.

### Individual-Level HMR

We used the Friston 24-parameter (F24) HM model (Friston et al., 1996) to perform individual-level HMR. This model includes six original HM parameters that characterize the current positions of the head, six HM parameters one time point before the original HM, which characterize past positions of the head, and the 12 corresponding square items representing non-linear influences (Friston et al., 1996).

As the current study focused on the effects of HM, we compared the results with and without HMR. For the case without HMR, the aforementioned covariates (WM, CSF, and SC regressors) were added together to the regression model, and then the residuals were used for the amplitude of fluctuation (AF, see below) calculation. It should be noted that amplitude of low frequency fluctuation (ALFF, Zang et al., 2007) was proposed to quantify the overall extent of slow fluctuation of rs-fMRI signals. In this study, we focused on both high- and low-frequency fluctuations, and therefore we used “AF” instead of “ALFF”. For the case with HMR, these HM regressors and the other covariates (WM, CSF, and SC regressors) were added together to the regression model, and then the residuals were used for the AF calculation.

### AF for fMRI Data

We calculated the high-frequency AF as in our previous work (Yuan et al., 2014). The procedures were as follows. First, the time series were transformed to frequency domain using a fast Fourier transform (FFT). The power spectrum was then obtained. The square root of the power spectrum was averaged across the frequency bands of interest (0.01–0.1 Hz, 0.1–0.15 Hz, 0.15–0.2 Hz, 0.2–0.25 Hz, 0.25–0.3 Hz, and 0.3–0.35 Hz). Consistent with our previous paper (Yuan et al., 2014), we defined the frequency bands between 0.01 and 0.1 Hz as low frequency. Those higher than 0.1 Hz were defined as high frequency. Because our previous study demonstrated that results below 0.35 Hz were more reproducible than those of the much higher-frequency bands, here we focused on the impact of HMR only on HFO results between 0.1 and 0.35 Hz.

For the purpose of standardization, the AF value of each voxel was divided by the mean AF within a “whole-brain” mask (Zang et al., 2007; Yan et al., 2013c; Yuan et al., 2014). This mask was obtained from the intersection of the non-zero voxels of all subjects’ normalized functional images and the whole-brain mask in the REST software. Before statistical analyses, the standardized AF maps were smoothed with a 6-mm full-width-half-maximum (FWHM) Gaussian kernel. The AF calculation was performed using the REST software, and the spatial smoothing was performed using SPM8. We calculated AF for data with and without individual-level HMR.

### Statistical Analyses

Paired *t*-tests were performed on the AF of each frequency band of interest (0.01–0.1 Hz, 0.1–0.15 Hz, 0.15–0.2 Hz, 0.2–0.25 Hz, 0.25–0.3 Hz, and 0.3–0.35 Hz) to reveal differences between EO and EC. The paired *t*-tests were implemented by the SPM8 package. For each comparison, smoothness (i.e., the full width at half maximum along the *x, y*, and *z* directions) was estimated based on the residual images after a general linear model (GLM) was fitted against the datasets (Bennett et al., 2009; Nichols, 2012). Then, the cluster size (*n*) needed for multiple comparison correction was determined by performing Monte Carlo simulations (Ledberg et al., 1998) using the Alphsim program in DPABI^4^ (Data Processing & Analysis of Brain Imaging). Note that the cluster-size threshold *n* is different for each comparison. This is because the estimated smoothness of the residual images were various across different frequency bands, and also different between the cases with and without HMR. A contiguity threshold of *n* contiguous voxels and voxel-level *p* < 0.01 were used as criteria for significant difference, corresponding to a corrected *p* < 0.05 within the “whole-brain” mask. Because these analyses were exploratory in nature, we did not perform the multiple comparison correction across different frequency bands.

### Group-Level HMR

Note that the aforementioned HMR was performed only at the individual level in which the HM time series were taken as regressors against the blood oxygen level dependent (BOLD) time series. In addition, we evaluated the effects of group-level HMR by taking the mean motion level of each single session as covariate for the group-level paired *t*-tests. A previous study demonstrated that group-level HMR could further reduce the residual relationships between motion and ALFF (Yan et al., 2013a). However, to what extent between-condition AF differences in low- and high-frequency bands are affected by group-level HMR is still unknown.

The mean motion level is usually quantified by mean framewise displacement (FD). There are different approaches to calculate the mean FD (Jenkinson et al., 2002; Power et al., 2012; Van Dijk et al., 2012). In this study, we used the method proposed by Jenkinson et al. (2002) because this method could best fit the mean FD of volume, while the other two methods either overestimated (Power et al., 2012) or underestimated (Van Dijk et al., 2012) the relationships (Yan et al., 2013a). The mean FD was characterized by the temporal mean of the volume-based FD time series. According to Jenkinson et al. (2002), the volume-based FD was obtained by:

(1) $$FD_{vol}(t)=\;\sqrt{\frac{1}{5}\;R^{2}Trace(A{\left(t\right)}^{T}A(t))\;+\;((b(t)\;+\;{\left(A(t)c\right)}^{T}(b(t)\;+\;(A(t)c)}$$

where *R* denotes the assumed radius specifying the head volume (usually *R* = 80 mm), superscript *T* indicates matrix transpose, *c* indicates the coordinates for the center of the volume, and *A* and *b* are defined as:

(2) $$\left[\begin{array}{cc} A(t) & b(t)\\ 0 & 0 \end{array}\right]\;=\;T(t)\;T{\left(t\;-\;1\right)}^{-1}\;-\;I$$

*T(t)* is the transformation matrix that transforms the volume at time point *t* to the position of the reference volume *I_0_* (the volume at the first time point)

(3) $$T(t)\;=\;\left[\begin{array}{cccc} 1 & 0 & 0 & x\\ 0 & 1 & 0 & y\\ 0 & 0 & 1 & z\\ 0 & 0 & 0 & 1 \end{array}\right]\;\left[\begin{array}{cccc} 1 & 0 & 0 & 0\\ 0 & \cos\alpha & \sin\alpha & 0\\ 0 & -\sin\alpha & \cos\alpha & 0\\ 0 & 0 & 0 & 1 \end{array}\right]\;\times \;\left[\begin{array}{cccc} \cos\beta & 0 & \sin\beta & 0\\ 0 & 1 & 0 & 0\\ -\sin\beta & 0 & \cos\beta & 0\\ 0 & 0 & 0 & 1 \end{array}\right]\;\left[\begin{array}{cccc} \cos\gamma & \sin\lambda & 0 & 0\\ -\sin\lambda & \cos\gamma & 0 & 0\\ 0 & 0 & 1 & 0\\ 0 & 0 & 0 & 1 \end{array}\right]$$

where *x, y, z* denote the translations, and α, β, γ denote rotations of the six HM parameters. *T(t)^-1^* is the rigid-body transformation of the reference volume *I_0_* to the position of the volume at time point *t*. The volume-based FD time series was calculated for each subject by DPABI. We compared results without HMR, with individual-level or group-level HMR, and with both individual- and group-level HMR by visual inspection.

## Results

### Power Spectra of HM Time Series

**FIGURE 2:**
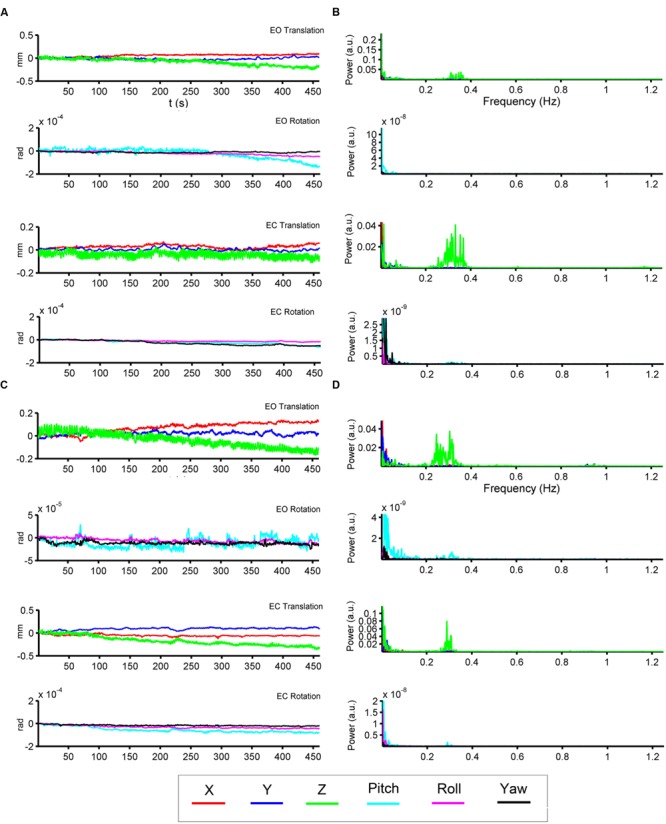
**Head motion (HM) time series and power spectra of two representative subjects.** The left panels **(A,C)** show the time series of six head motion parameters [translation: *x* (red), *y* (blue), *z* (green); rotations: pitch (cyan), roll (magenta), yaw (black)] in both EO and EC conditions. The right panels **(B,D)** show the corresponding power spectra.

We show the HM time series and their corresponding power spectra from two representative subjects (**Figures 2A–D**). In both subjects, most signal power was located in the low-frequency range in both translation and rotation directions. Moreover, we also observed prominent signal power around 0.3 Hz in the *z* direction of the translation (**Figures 2B,D**).

**FIGURE 3:**
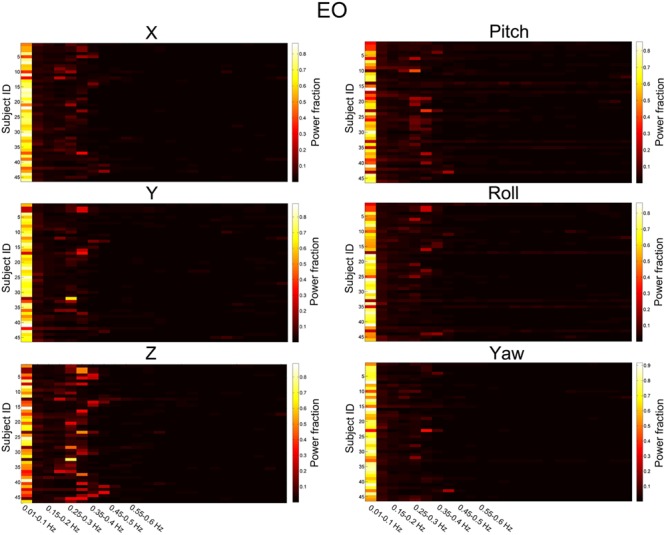
**Power fraction of HM time series.** The power spectra of HM time series were summarized for all of the subjects for the EO state. Each panel represents one direction of HM. For each panel, each row represents the power fraction (i.e., the ratio of sub-band power to the total power) of HM time series for one subject. Apparent high-frequency (∼0.2–0.4 Hz) components of the HM trajectories could be observed in almost all subjects.

**FIGURE 4:**
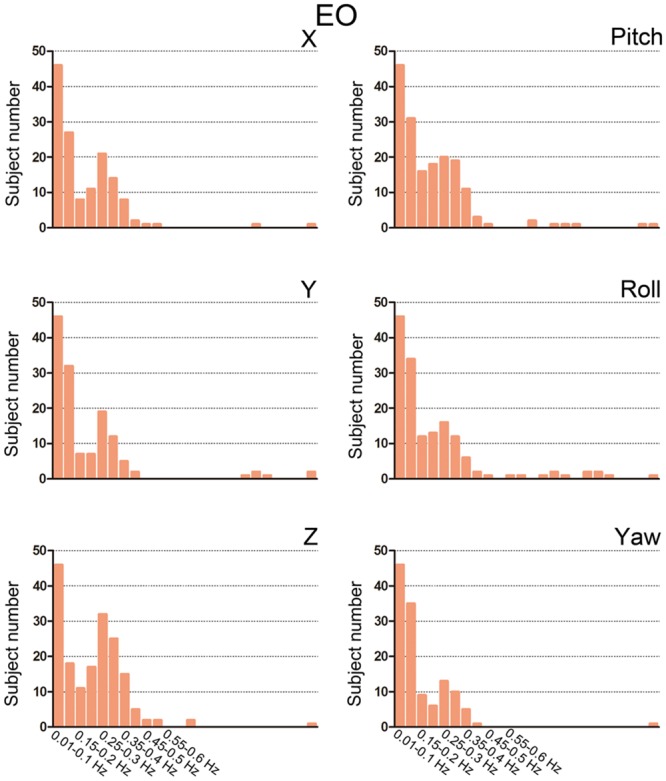
**Bar graphs showing the number of subjects with prominent high-frequency HM.** For each sub-band, we counted the number of subjects whose power fraction was larger than 1/24 total power for the EO state. We used the threshold 1/24 because there are 23 high-frequency sub-bands between 0.1 and 1.25 Hz when the step is 0.05 Hz. If the high-frequency HM was not large enough, then the power should be equally distributed between 0.1 and 1.25 Hz.

We then summarized the HM power spectra for all of the subjects and all of the directions as follows. We calculated the fraction of sub-band (i.e., 0.01–0.1 Hz, 0.1–0.15 Hz, 0.15–0.2 Hz, 1.2–1.25 Hz) power to the total power. Prominent high-frequency HM was a common phenomenon for the majority of subjects (more than 60% for the *z* direction, **Figures 3** and **4**). However, the power was located primarily in the frequency range below 0.4 Hz. High-frequency HM was evident for the *z* direction of translation and pitch for the rotation, relative to the other directions (**Figure 3**). This trend was generally the same for EO and EC conditions (The results for EC are presented in the Supplementary Materials). These results suggest that fast oscillations exist in the HM trajectory, and such rapid head movements may bias HFO results.

### The Results of Individual-Level HMR

**FIGURE 5:**
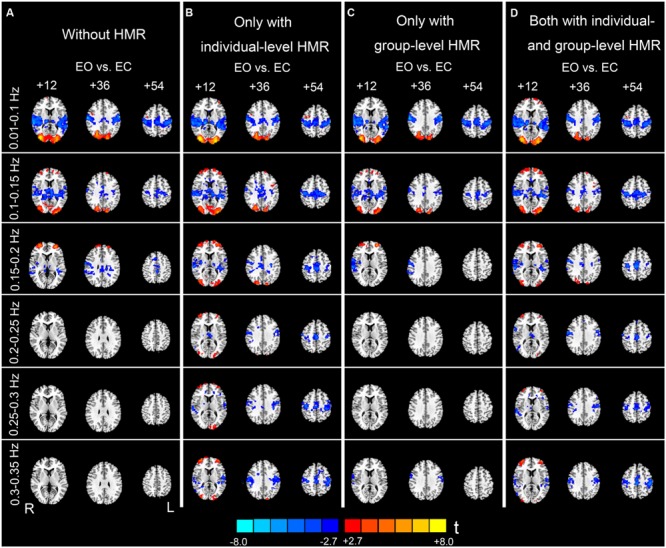
**Paired *t*-test results of the AF differences between EO and EC.** Column **(A)** shows results without HMR. Column **(B)** shows results with individual-level HMR. Column **(C)** shows results with group-level HMR. Column **(D)** shows results with both individual- and group-level HMR (*p* < 0.05, corrected). Warm color indicates higher AF in EO compared with EC, and cold color indicates the opposite. The left side of the figure corresponds to the right side of the brain.

At 0.01–0.1 Hz, the results with and without HMR were very similar. For both types of results, significantly increased AF in EO was observed in the bilateral middle occipital gyrus (MOG). The regions showing decreased AF in EO compared with EC were mainly in the bilateral primary auditory cortex (PAC), primary sensorimotor cortex (PSMC), supplementary motor area (SMA), and thalamus (*p* < 0.05, corrected, **Figures 5A,B**).

For the high-frequency results, we observed clear differences between results with and without individual-level HMR. In particular, in the frequency bands between 0.2 and 0.35 Hz, more regions in the bilateral PSMC and PAC could be identified with HMR than without. Such results suggest that HMR can facilitate detection of between-condition differences, which may be achieved by suppressing HM artifacts. At 0.25–0.3 Hz, more regions in the prefrontal cortex could be identified in results without HMR. However, we speculated that such results may reflect HM artifacts, and this bias might be corrected by HMR. Notably, our results (**Figures 3** and **4**) indicated large variability of high-frequency HM across subjects. Although high-frequency HM was evident for some subjects, its effects were weak for other subjects, which suggests that the influence of high-frequency HM is not a systematic effect. Therefore, we did not perform a statistical comparison between results with and without individual-level HMR.

### The Results of Group-Level HMR

Although individual-level HMR can facilitate detection of the symmetrical pattern in the PSMC and PAC in high-frequency sub-bands (0.2–0.35 Hz, **Figures 5A,B**), the results with group-level HMR differed from those without HMR only in a few foci in the PSMC (e.g., 0.3–0.35 Hz, **Figures 5A,C**). The results with both individual- and group- level HMR were very similar to those with only individual-level HMR (**Figures 5B,D**).

**FIGURE 6:**
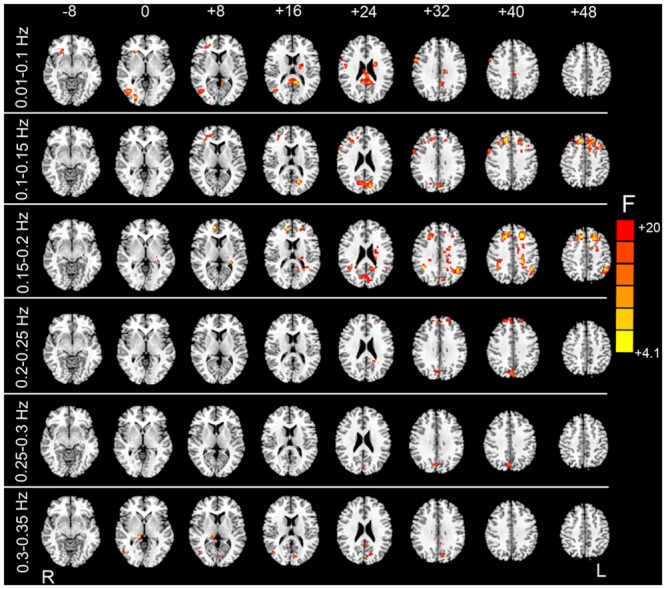
***F* test results showing the HM-related effects.** By taking the mean FD as a covariate in the paired *t*-test GLM, we examined whether mean FD could account for some inter-subject variance. After individual-level HMR, motion-related variance was still significant in the prefrontal cortex, visual cortex, and white matter (WM) (*p* < 0.05, corrected).

Although group-level HMR seems to have little impact on detection of between-condition differences, we found that the mean FD significantly accounted for the inter-subject variance mainly for the sub-bands between 0.01 and 0.25 Hz (**Figure 6**). These results suggest that even after individual-level HMR, the residual motion effect (at the group level) was still pronounced in HFOs.

## Discussion

In this study, we investigated high-frequency properties of HM and influences of HMR on HFO results. First, we found that high-frequency HM is a relatively common phenomenon. We then showed that individual-level HMR could successfully suppress motion artifacts and revealed more between-condition differences in HFOs. Moreover, we showed that a group-level FD regressor could account for marked inter-subject variance in HFOs even after performing individual-level HMR.

### Spectral Characteristics of HM

The HM effect on HFOs is related to its properties. Previous studies have revealed various features of HM including temporal, spatial, and distance-dependent impacts on fMRI data (see review by Power et al., 2015), whereas no study has examined the spectral characteristics of HM. In this study, we used a fast sampling technique and examined the power spectra of the HM time series. We found that, although the HM process is dominated by low-frequency fluctuations, there was a considerable amount of high-frequency power for the majority of subjects. Such high-frequency components were dominant at 0.2–0.4 Hz.

The frequency band of high-frequency HM overlaps with the respiratory process. However, we speculate that the two processes (i.e., HM and breathing) may have different impacts on fMRI data. Even if we regressed out the covariates from WM, CSF, and SC, which were assumed to represent physiological activity-induced noise, there were still clear differences in the spatial patterns between results with and without individual-level HMR. Only when individual-level HMR was performed, motion artifacts in high-frequency bands could be efficiently reduced and more between-condition differences could be revealed (**Figure 5**). The confounding effect of motion is particularly evident at tissue interfaces such as gray/WM boundaries, around large blood vessels, and at the edges of the brain, while respiratory-induced noise is pronounced in ventricles and veins (Birn et al., 2006; Lund et al., 2006). Thus, although the two physiological processes have overlapping high-frequency bands, their impacts may be partly independent. However, because we did not directly record the respiratory signal, the aforementioned hypothesis should be tested in future studies.

### The Influences of Individual-Level HMR

The effect of HM on the fMRI time series is complex, and various HMR strategies at both individual- and group-levels have been proposed (See the review by Power et al., 2015). The substantial benefits for controlling the HM artifact using numerous proposed HM correction strategies have been revealed by previous studies (Power et al., 2012, 2014; Satterthwaite et al., 2012, 2013; Van Dijk et al., 2012; Yan et al., 2013a,b). Here we showed that individual-level HMR using the F24 model revealed more high-frequency AF differences in the bilateral PSMC and PAC, which were rarely observed when we did not perform HMR. Our findings suggest the benefit and necessity of individual-level HMR for HFO studies.

We believe that the AF differences in the PSMC and PAC are not artifacts introduced by HMR but may have a neural basis. The differences in ALFF in the bilateral PSMC and PAC have been consistently reported in previous EO-EC studies that used different MRI scanners, parameters, and data processing strategies (Yan et al., 2009; Liu et al., 2013; Yuan et al., 2014; Zou et al., 2015). Changes in spontaneous brain activity in these two regions may reflect cross-modal inhibition (Laurienti et al., 2002). Here, the observed high-frequency differences may be an extension of the low-frequency results.

The F24 HM model we used has been shown to perform better than other low-order models (Satterthwaite et al., 2013; Yan et al., 2013a,b). Although we did not perform a comparison between the high- (e.g., F24 regression model used in the current study) and low-order (e.g., commonly used rigid-body 6-parameter model) approaches, we believe that the F24 model is superior to the low-order model in that it takes both the temporal and non-linear effects of HM into consideration. As shown by Satterthwaite et al. (2013) and Power et al. (2014), the motion artifact induced by a sudden HM could last for several seconds after the motion ends, which indicates the pronounced temporal effect of HM. Moreover, compared with the regression model without the squared items, significantly larger variances could be accounted for by those regression models with squared items (Satterthwaite et al., 2013; Yan et al., 2013a), which suggests the existence of a non-linear effect of HM on the BOLD time series.

### The Influences of Group-Level HMR

The primary aim of group-level motion correction is to reduce motion-related group differences (Satterthwaite et al., 2012; Van Dijk et al., 2012; Yan et al., 2013a; Power et al., 2014). It has been shown that, for subjects with high motion amplitude, the combination of group-level and individual-level HMR could greatly reduce motion artifacts compared with individual-level HMR. In the current study, however, we found that group-level HMR had little impact on the between-condition HFO results irrespective of whether or not individual-level HMR was performed (**Figure 4**). We speculate that this may be due to the fact that the overall HM of the subjects we studied was not very large, as the mean FD for EO was 0.033 (*SD* = 0.011) and 0.029 (*SD* = 0.008) for EC. Nevertheless, we also performed an *F* test on the HM covariate and found pronounced motion-related inter-subject variance (**Figure 6**), which suggests that motion effects at the group level cannot be ignored. Further studies including subjects with larger HM are needed to understand comprehensively the influences of group-level HMR.

### Limitations

In this study, we used a relative short TE (15 ms) which is not commonly used in BOLD-fMRI studies. This is because the present study aimed to investigate the HFOs using fast sampling rate and within the whole brain. However, for the conventional techniques, increasing TR will be at the cost of losing brain coverage. In order to meet both criteria, fast sampling rate and large brain coverage, we had to increase the slice thickness and shorten the TE, as there is a trade-off between the TE and the maximum number of slices for our scanner. Too short TE may not be optimal to distinguish among different types of tissues. In contrast, recently developed fast-sampling techniques (Hennig et al., 2007; Feinberg et al., 2010; Boyacioglu and Barth, 2013) have the capacity of acquiring whole brain data within less than 1 s, most importantly, with high spatial resolution and good contrast. Although the between-condition differences observed in the present study were highly consistent with previous findings (Yan et al., 2009; Liu et al., 2013; Yuan et al., 2014; Zou et al., 2015), further HFO studies combined with fast-sampling sequences are suggested to reveal the high-frequency properties of rs-fMRI signals.

One concern that must be noted is the neurobiological significance of HM. The present study was performed only on the healthy subjects. Several studies have revealed that HM may reflect a trait effect (Kong et al., 2014; Zeng et al., 2014) or motion-related neural activity (Yan et al., 2013a; Pujol et al., 2014). It can be speculated that HM in patients (e.g., attention-deficit hyperactivity disorder) may contain pathological information. In such cases, motion covariates are likely to be collinear with the effects of interest, and thus HMR will probably remove the effects under investigation. Although the current work suggests that HMR should be considered as a critical step for HFO studies, caution is still needed when atypical participants are studied.

## Conclusion

In summary, our results indicate that conspicuous HFOs exist in HM trajectories. Individual-level HMR can facilitate detection of between-condition differences in high-frequency bands and reduce the artifacts. Mean motion level still accounts for large inter-subject variance even after individual-level HMR is performed. Thus, HM artifacts cannot be ignored for HFO studies. We highly recommend that both individual- and group- level approaches should be employed.

## Author Contributions

D-QL and Y-FZ conceived and designed the experiment. B-KY and D-QL performed the experiment. B-KY and D-QL analyzed the data. B-KY, Y-FZ, and D-QL wrote the paper.

## Conflict of Interest Statement

The authors declare that the research was conducted in the absence of any commercial or financial relationships that could be construed as a potential conflict of interest.

## Footnotes

1 http://fcon_1000.projects.nitrc.org/indi/IndiRetro.html

2 http://www.fil.ion.ucl.ac.uk/spm

3 www.restfmri.net

4 http://rfmri.org/dpabi

## References

[B1] Baliki M. N. Baria A. T. Apkarian A. V. (2011). The cortical rhythms of chronic back pain. *J. Neurosci.* 31 13981–13990. 10.1523/JNEUROSCI.1984-11.2011 21957259 PMC3214084

[B2] Baria A. T. Baliki M. N. Parrish T. Apkarian A. V. (2011). Anatomical and functional assemblies of brain BOLD oscillations. *J. Neurosci.* 31 7910–7919. 10.1523/JNEUROSCI.1296-11.2011 21613505 PMC3114444

[B3] Bennett C. M. Wolford G. L. Miller M. B. (2009). The principled control of false positives in neuroimaging. *Soc. Cogn. Affect. Neurosci.* 4 417–422. 10.1093/scan/nsp053 20042432 PMC2799957

[B4] Birn R. M. Diamond J. B. Smith M. A. Bandettini P. A. (2006). Separating respiratory-variation-related neuronal-activity-related fluctuations in fluctuations from fMRI. *Neuroimage* 31 1536–1548. 10.1016/j.neuroimage.2006.02.048 16632379

[B5] Biswal B. Yetkin F. Z. Haughton V. M. Hyde J. S. (1995). Functional connectivity in the motor cortex of resting human brain using echo-planar MRI. *Magn. Reson. Med.* 34 537–541. 10.1002/mrm.1910340409 8524021

[B6] Boubela R. N. Kalcher K. Huf W. Kronnerwetter C. Filzmoser P. Moser E. (2013). Beyond noise: using temporal ICA to extract meaningful information from high-frequency fMRI signal fluctuations during rest. *Front. Hum. Neurosci.* 7:168 10.3389/fnhum.2013.00168 PMC3640215 23641208

[B7] Boyacioglu R. Barth M. (2013). Generalized INverse imaging (GIN): ultrafast fMRI with physiological noise correction. *Magn. Reson. Med.* 70 962–971. 10.1002/mrm.24528 23097342

[B8] Boyacioglu R. Beckmann C. F. Barth M. (2013). An investigation of RSN frequency spectra using ultra-fast generalized inverse imaging. *Front. Hum. Neurosci.* 7:156 10.3389/fnhum.2013.00156 PMC3632876 23630487

[B9] Cordes D. Haughton V. M. Arfanakis K. Carew J. D. Turski P. A. Moritz C. H. (2001). Frequencies contributing to functional connectivity in the cerebral cortex in “resting-state” data. *AJNR Am. J. Neuroradiol.* 22 1326–1333. 11498421 PMC7975218

[B10] Cordes D. Haughton V. M. Arfanakis K. Wendt G. J. Turski P. A. Moritz C. H. (2000). Mapping functionally related regions of brain with functional connectivity MR imaging. *AJNR Am. J. Neuroradiol.* 21 1636–1644. 11039342 PMC8174861

[B11] Feinberg D. A. Moeller S. Smith S. M. Auerbach E. Ramanna S. Gunther M. (2010). Multiplexed echo planar imaging for sub-second whole brain FMRI and fast diffusion imaging. *PLoS ONE* 5:e15710 10.1371/journal.pone.0015710 PMC3004955 21187930

[B12] Friston K. J. Williams S. Howard R. Frackowiak R. S. Turner R. (1996). Movement-related effects in fMRI time-series. *Magn. Reson. Med.* 35 346–355. 10.1002/mrm.1910350312 8699946

[B13] Gohel S. R. Biswal B. B. (2014). Functional integration between brain regions at rest occurs in multiple-frequency bands. *Brain Connect.* 5 23–34. 10.1089/brain.2013.0210 24702246 PMC4313418

[B14] Greicius M. D. Krasnow B. Reiss A. L. Menon V. (2003). Functional connectivity in the resting brain: a network analysis of the default mode hypothesis. *Proc. Natl. Acad. Sci. U.S.A.* 100 253–258. 10.1073/pnas.0135058100 12506194 PMC140943

[B15] Hennig J. Zhong K. Speck O. (2007). MR-Encephalography: fast multi-channel monitoring of brain physiology with magnetic resonance. *Neuroimage* 34 212–219. 10.1016/j.neuroimage.2006.08.036 17071111

[B16] Hong J. Y. Kilpatrick L. A. Labus J. Gupta A. Jiang Z. Ashe-McNalley C. (2013). Patients with chronic visceral pain show sex-related alterations in intrinsic oscillations of the resting brain. *J. Neurosci.* 33 11994–12002. 10.1523/JNEUROSCI.5733-12.2013 23864686 PMC3713732

[B17] Jenkinson M. Bannister P. Brady M. Smith S. (2002). Improved optimization for the robust and accurate linear registration and motion correction of brain images. *Neuroimage* 17 825–841. 10.1006/nimg.2002.1132 12377157

[B18] Kalcher K. Boubela R. N. Huf W. Bartova L. Kronnerwetter C. Derntl B. (2014). The spectral diversity of resting-state fluctuations in the human brain. *PLoS ONE* 9:e93375 10.1371/journal.pone.0093375 PMC3984093 24728207

[B19] Kong X. Z. Zhen Z. Li X. Lu H. H. Wang R. Liu L. (2014). Individual differences in impulsivity predict head motion during magnetic resonance imaging. *PLoS ONE* 9:e104989 10.1371/journal.pone.0104989 PMC4141798 25148416

[B20] Laurienti P. J. Burdette J. H. Wallace M. T. Yen Y. F. Field A. S. Stein B. E. (2002). Deactivation of sensory-specific cortex by cross-modal stimuli. *J. Cogn. Neurosci.* 14 420–429. 10.1162/089892902317361930 11970801

[B21] Ledberg A. Akerman S. Roland P. E. (1998). Estimation of the probabilities of 3D clusters in functional brain images. *Neuroimage* 8 113–128. 10.1006/nimg.1998.0336 9740755

[B22] Lee H. L. Zahneisen B. Hugger T. LeVan P. Hennig J. (2013). Tracking dynamic resting-state networks at higher frequencies using MR-encephalography. *Neuroimage* 65 216–222. 10.1016/j.neuroimage.2012.10.015 23069810

[B23] Liu D. Q. Dong Z. Y. Zuo X. N. Wang J. Zang Y. F. (2013). Eyes-open/eyes-closed dataset sharing for reproducibility evaluation of resting state fmri data analysis methods. *Neuroinformatics* 11 469–476. 10.1007/s12021-013-9187-0 23836389

[B24] Lowe M. J. Mock B. J. Sorenson J. A. (1998). Functional connectivity in single and multislice echoplanar imaging using resting-state fluctuations. *Neuroimage* 7 119–132. 10.1006/nimg.1997.0315 9558644

[B25] Lund T. E. Madsen K. H. Sidaros K. Luo W. L. Nichols T. E. (2006). Non-white noise in fMRI: does modelling have an impact? *Neuroimage* 29 54–66. 10.1016/j.neuroimage.2005.07.005 16099175

[B26] Malinen S. Vartiainen N. Hlushchuk Y. Koskinen M. Ramkumar P. Forss N. (2010). Aberrant temporal and spatial brain activity during rest in patients with chronic pain. *Proc. Natl. Acad. Sci. U.S.A.* 107 6493–6497. 10.1073/pnas.1001504107 20308545 PMC2852014

[B27] Nichols T. E. (2012). Multiple testing corrections, nonparametric methods, and random field theory. *Neuroimage* 62 811–815. 10.1016/j.neuroimage.2012.04.014 22521256

[B28] Otti A. Guendel H. Wohlschlager A. Zimmer C. Noll-Hussong M. (2013). Frequency shifts in the anterior default mode network and the salience network in chronic pain disorder. *BMC Psychiatry* 13:84 10.1186/1471-244X-13-84 PMC3616999 23497482

[B29] Power J. D. Barnes K. A. Snyder A. Z. Schlaggar B. L. Petersen S. E. (2012). Spurious but systematic correlations in functional connectivity MRI networks arise from subject motion. *Neuroimage* 59 2142–2154. 10.1016/j.neuroimage.2011.10.018 22019881 PMC3254728

[B30] Power J. D. Mitra A. Laumann T. O. Snyder A. Z. Schlaggar B. L. Petersen S. E. (2014). Methods to detect, characterize, and remove motion artifact in resting state fMRI. *Neuroimage* 84 320–341. 10.1016/j.neuroimage.2013.08.048 23994314 PMC3849338

[B31] Power J. D. Schlaggar B. L. Petersen S. E. (2015). Recent progress and outstanding issues in motion correction in resting state fMRI. *Neuroimage* 105 536–551. 10.1016/j.neuroimage.2014.10.044 25462692 PMC4262543

[B32] Pujol J. Macià D. Blanco-Hinojo L. Martínez-Vilavella G. Sunyer J. de la Torre R. (2014). Does motion-related brain functional connectivity reflect both artifacts and genuine neural activity? *Neuroimage* 101 87–95. 10.1016/j.neuroimage.2014.06.065 24999036

[B33] Satterthwaite T. D. Elliott M. A. Gerraty R. T. Ruparel K. Loughead J. Calkins M. E. (2013). An improved framework for confound regression and filtering for control of motion artifact in the preprocessing of resting-state functional connectivity data. *Neuroimage* 64 240–256. 10.1016/j.neuroimage.2012.08.052 22926292 PMC3811142

[B34] Satterthwaite T. D. Wolf D. H. Loughead J. Ruparel K. Elliott M. A. Hakonarson H. (2012). Impact of in-scanner head motion on multiple measures of functional connectivity: relevance for studies of neurodevelopment in youth. *Neuroimage* 60 623–632. 10.1016/j.neuroimage.2011.12.063 22233733 PMC3746318

[B35] Song X. W. Dong Z. Y. Long X. Y. Li S. F. Zuo X. N. Zhu C. Z. (2011). REST: a toolkit for resting-state functional magnetic resonance imaging data processing. *PLoS ONE* 6:e25031 10.1371/journal.pone.0025031 PMC3176805 21949842

[B36] Van Dijk K. R. Sabuncu M. R. Buckner R. L. (2012). The influence of head motion on intrinsic functional connectivity MRI. *Neuroimage* 59 431–438. 10.1016/j.neuroimage.2011.07.044 21810475 PMC3683830

[B37] Wang J. Zhang Z. Ji G.-J. Xu Q. Huang Y. Wang Z. (2015). Frequency-specific alterations of local synchronization in idiopathic generalized epilepsy. *Medicine* 94:e1374 10.1097/MD.0000000000001374 PMC4616718 26266394

[B38] Yan C. Liu D. He Y. Zou Q. Zhu C. Zuo X. (2009). Spontaneous brain activity in the default mode network is sensitive to different resting-state conditions with limited cognitive load. *PLoS ONE* 4:e5743 10.1371/journal.pone.0005743 PMC2683943 19492040

[B39] Yan C. G. Cheung B. Kelly C. Colcombe S. Craddock R. C. Di Martino A. (2013a). A comprehensive assessment of regional variation in the impact of head micromovements on functional connectomics. *Neuroimage* 76 183–201. 10.1016/j.neuroimage.2013.03.004 23499792 PMC3896129

[B40] Yan C. G. Craddock R. C. He Y. Milham M. P. (2013b). Addressing head motion dependencies for small-world topologies in functional connectomics. *Front. Hum. Neurosci.* 7:910 10.3389/fnhum.2013.00910 PMC3872728 24421764

[B41] Yan C. G. Craddock R. C. Zuo X. N. Zang Y. F. Milham M. P. (2013c). Standardizing the intrinsic brain: towards robust measurement of inter-individual variation in 1000 functional connectomes. *Neuroimage* 80 246–262. 10.1016/j.neuroimage.2013.04.081 23631983 PMC4074397

[B42] Yu X. Yuan B. Cao Q. An L. Wang P. Vance A. (2015). Frequency-specific abnormalities in regional homogeneity among children with attention deficit hyperactivity disorder: a resting-state fMRI study. *Sci. Bull.* 61 682–692. 10.1007/s11434-015-0823-y

[B43] Yuan B.-K. Wang J. Zang Y.-F. Liu D.-Q. (2014). Amplitude differences in high-frequency fMRI signals between eyes open and eyes closed resting states. *Front. Hum. Neurosci.* 8:503 10.3389/fnhum.2014.00503 PMC4086401 25071530

[B44] Yue Y. Jia X. Hou Z. Zang Y. Yuan Y. (2015). Frequency-dependent amplitude alterations of resting-state spontaneous fluctuations in late-onset depression. *BioMed Res. Int.* 2015:9 10.1155/2015/505479 PMC4331395 25705666

[B45] Zang Y. F. He Y. Zhu C. Z. Cao Q. J. Sui M. Q. Liang M. (2007). Altered baseline brain activity in children with ADHD revealed by resting-state functional MRI. *Brain Dev.* 29 83–91. 10.1016/j.braindev.2006.07.002 16919409

[B46] Zeng L. L. Wang D. Fox M. D. Sabuncu M. Hu D. Ge M. (2014). Neurobiological basis of head motion in brain imaging. *Proc. Natl. Acad. Sci. U.S.A.* 111 6058–6062. 10.1073/pnas.1317424111 24711399 PMC4000812

[B47] Zou Q. Yuan B.-K. Gu H. Liu D. Wang D. J. Gao J.-H. (2015). Detecting static and dynamic differences between eyes-closed and eyes-open resting states using ASL and BOLD fMRI. *PLoS ONE* 10:e0121757 10.1371/journal.pone.0121757 PMC4376626 25816237

