# Supplementary material for: Influences of Head Motion Regression on High-Frequency Oscillation Amplitudes of Resting-State fMRI Signals
Source: Front Hum Neurosci. 2016 May 26;10:243. doi: 10.3389/fnhum.2016.00243 (PMC4881380; doi:10.3389/fnhum.2016.00243)
Supplement: Supplementary file 1 [file Image_1.PDF]

Influences of head motion regression on high-frequency oscillation amplitude of resting-state fMRI signals

Bin-Ke Yuan[1,2], Yu-Feng Zang[1,2], Dong-Qiang Liu[3*]

[1] Center for Cognition and Brain Disorders, Hangzhou Normal University, Hangzhou, China

[2] Zhejiang Key Laboratory for Research in Assessment of Cognitive Impairments, Hangzhou Normal University, Hangzhou, China

[3] Research Center of Brain and Cognitive Neuroscience, Liaoning Normal University, Dalian, China

***Correspondence:** Dong-Qiang Liu (charlesliu116@gmail.com)

Supplementary Figures

Figure S1. **Power fraction of HM time series for EC state**. The power spectrum of HM time series were summarized for all the subjects for EC state. Each panel represents one direction of HM. For each panel, each row represents the power fraction (i.e., the ratio of sub-band power to the total power) of HM time series for one subject. Apparent high-frequency (~0.2-0.4 Hz) components of the HM trajectories could be observed in almost all subjects.

Figure S2. Barplots showing the number of subjects with prominent high-frequency HM. For each sub-band, we counted the number of subjects whose power fraction was larger than 1/24 of total power for the EC state. We used the threshold 1/24 because there are totally 23 high-frequency sub-bands between 0.1 and 1.25 Hz, given the step is 0.05 Hz. If the high-frequency HM was not large enough, then the power should be equally distributed between 0.1-1.25 Hz.

Supplementary Figures

Figure S1

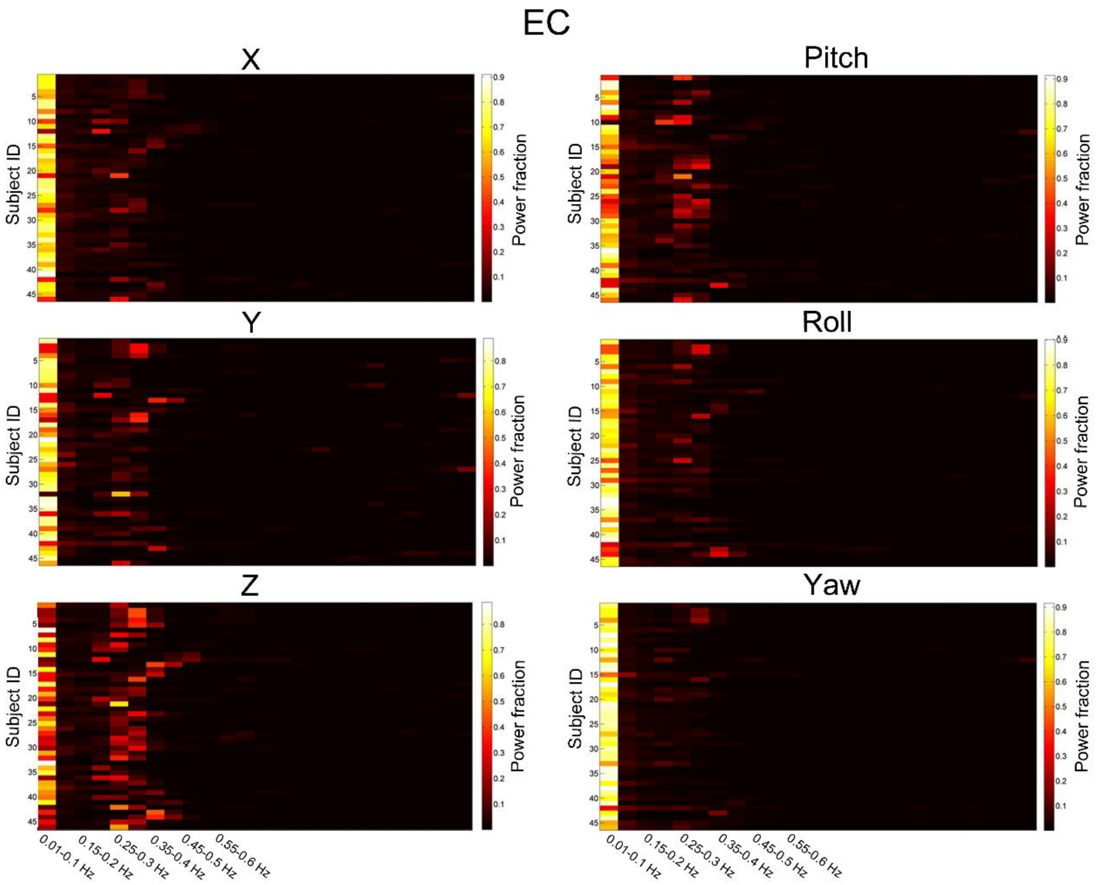

Figure S1. **Power fraction of HM time series for EC state**. The power spectrum of HM time series were summarized for all the subjects for EC state. Each panel represents one direction of HM. For each panel, each row represents the power fraction (i.e., the ratio of sub-band power to the total power) of HM time series for one subject. Apparent high-frequency (~0.2-0.4 Hz) components of the HM trajectories could be observed in almost all subjects.

Figure S2

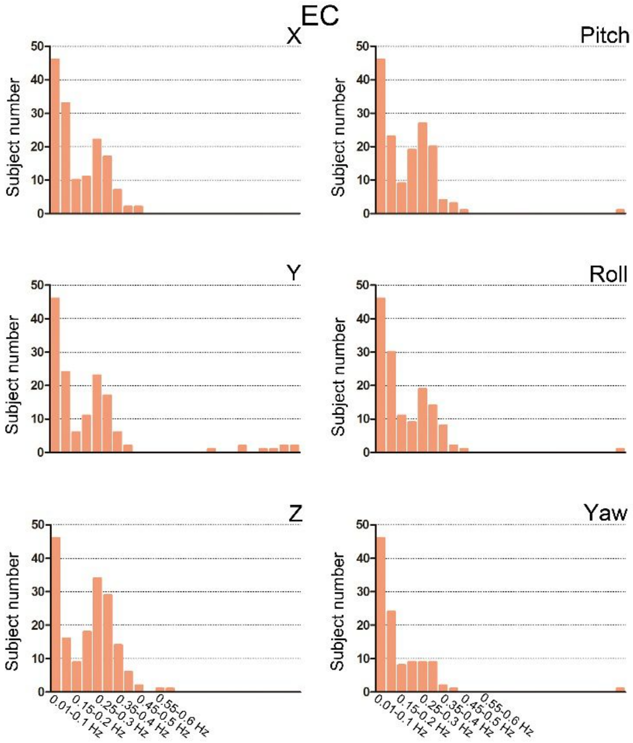

Figure S2. Barplots showing the number of subjects with prominent high-frequency HM. For each sub-band, we counted the number of subjects whose power fraction was larger than 1/24 of total power for the EC state. We used the threshold 1/24 because there are totally 23 high-frequency sub-bands between 0.1 and 1.25 Hz, given the step is 0.05 Hz. If the high-frequency HM was not large enough, then the power should be equally distributed between 0.1-1.25 Hz.
